# An Unusual Instance of Poisoning with Argyrol Injected into the Deep Urethra and Bladder

**Published:** 1910-07

**Authors:** Edgar G. Ballenger

**Affiliations:** Atlanta, Ga.


### AN UNUSUAL INSTANCE OF POISONING WITH ARGYROL INJECTED INTO THE DEEP URETHRA AND BLADDER.

BY EDGAR G. BALLENGER, M. D., ATLANTA, GA.

Argyrol being considered a non-toxic remedy, I desire to report the following history in which its poisonous effects were alarmingly shown:

Mr. A, age 35, married, family history and past history negative; general health good.

The patient came to me for the treatment of a severe acute urethro-cysto-prostatitis of gonorrhoeal origin. There seemed to be nothing unusual in the character of the disease on the anatomy of the genito-urinary organs.

He was given an urethral and bladder irrigation with 1 to 6000 potassium permanganate in hot normal salt solution, which was administered without difficulty.

In order that the inflamed bladder should receive the full benefit of the injection treatment to be administered by the patient, he was instructed to procure a two-ounce syringe with a blunt point and after urination to inject a syringeful of a three per cent. solution of argyrol into the bladder once or twice daily and to retain the fluid until he desired to urinate.

Fortunately for the patient, the first treatment was taken at his home in his bath-room, where his wife came to the rescue with a very hot bath, or the result may have been fatal.

As soon as the argyrol solution reached the bladder a slight spasm of the deep urethra prevented its expulsion, as in two minutes he attempted to urinate on account of the pain he experienced in the bladder. Within five minutes the penis and foreskin were swollen as large as his arm and his entire body, especially his face, began to swell, and in ten minutes his wife states he was so bloated she would not have recognized him. While in the bath tub he had a severe chill which recurred several times during the following six hours. His temperature, he states, was very high although it was not determined with a thermometer. His lips were enormously swollen and did not become normal for several days. His eyes protruded in such a manner as to give him a ghastly appearance. The tongue, lips, gums and throat were very elematous and rendered breathing difficult. There was a peculiar astringent metallic taste in his mouth. After a little while the argyrol began to pass but was not thoroughly voided until two hours later. The patient was more or less dazed during the first part of the attack and suffered from a severe headache, depression, and numbness over the body for two days.

Having used argyrol extensively since its introduction and never having heard of its producing such a train of toxic symptoms, I felt inclined to believe they were perhaps due more to a urinary chill or some such condition than to the argyrol; nevertheless, I told the patient to discontinue the deep injection of argyrol but to take the injections in the anterior urethra. In about four days he was feeling so well he could see no reason why the deeper parts should not receive the benefit of the treatment also, and he proceeded to inject one-half of the large syringeful into the bladder, but taking the precaution to have a tub of hot water drawn. Immediately an attack was precipitated which was worse than the previous one, the patient falling to the floor and for a time being unconscious, and when he became conscious, was unable to speak for about 15 minutes.

Another attack two weeks later was brought on when, in spite of instruction to the contrary, he injected with a small syringe about 2 drachms of a 4% solution of argyrol into the deep urethra and bladder.

Still a fourth, but slight attack, followed a two copious anterior injection three weeks later when a small amount of the solution was felt to pass into the deep urethra; most of this was quickly voided but within five minutes the penis had swollen to twice its normal size. No other untoward symptoms followed this attack.

There was no abscess cavity or diverticulum into which the solution went and in spite of the systemic effect there was no urethral or bladder irritation produced by the argyrol and the patient has made a very satisfactory recovery.

I am unable to give a reason for the fact that the toxic symptoms should follow when the solution entered the deep urethra and yet not the slightest amount be produced when only the anterior urethra was treated.

Permanganate irrigations of the bladder produced no irritation or ill effects, and have been continued throughout the treatment.

I have seen two patients with idiosyncrasies for argyrol when ejected into the urethra, but the effect was only a local irritative one, and merely required the discontinuance or a change of injection.

The patient states that very hot baths afforded much relief from the toxic symptoms.

